# Safety analysis of raltegravir/truvada regimen in HIV/HCV co-infected patients without switchback after HCV treatment

**DOI:** 10.7448/IAS.17.4.19644

**Published:** 2014-11-02

**Authors:** Robert Ehret, Hauke Walter, Patrick Ingiliz, Axel Baumgarten, Martin Obermeier, Ivanka Krznaric

**Affiliations:** 1 Laboratory, MIB, Berlin, Germany; 2 Out Patients Clinic, MIB, Berlin, Germany

## Abstract

**Introduction:**

Due to drug-drug interactions of HIV- and HCV-specific antivirals when initiating an HCV-therapy, the antiretroviral therapy (ART) often has to be changed. The spectrum of applicable antiretrovirals is small, therefore many patients were switched to raltegravir/truvada (RAL/TVD) in our cohort. Due to the relatively low genetic barrier of RAL, this regimen may be endangered to fail, if the NRTI backbone is not fully active because of pre-existing NRTI resistance. We investigated the long-term follow-up and safety of RAL/TVD in co-infected patients after hepatitis C virus (HCV) therapy was stopped and the protective antiretroviral effect of interferon ended.

**Materials and Methods:**

Twenty patients initiated a direct-acting antiviral (DAA) containing HCV therapy (8x faldaprevir, 6x telaprevir, 2x daclatasvir and 4x simeprevir) between 11/2011 and 01/2013. Seventeen were switched to RAL/TVD, three patients were not treated before, but started with the regimen. Diagnosis of HIV infection was dated between 1985 and 2010. The HI-viral suppression was monitored retrospectively to date.

**Results:**

Thirteen of the twenty patients (65%) remained on RAL/TVD after finishing HCV treatment, for seven patients, no data about their ART continuation was available, after HCV therapy had stopped. All remaining thirteen patients showed an HI-viral load below detection limit up to date (for 15 to 22 months, median 20 months). Only for four patients, historic resistance data were available but none showed NRTI mutations.

**Conclusions:**

Switch to RAL/TVD as HIV ART due to initiating HCV therapy was safe for the observed small cohort even in long-term follow-up without switchback or a second ART switch. However, resistance data for the cohort was little, showing no NRTI mutations, indicating a relatively safe setting. Since no further data is available, physicians should keep in mind ART history, historical therapy failure and HIV-resistance while switching ART to treat HCV in co-infected patients. Further investigation in larger cohorts is needed, especially thinking of upcoming interferon-free HCV regimen in heavily pre-treated co-infected patients.

